# Evaluation of the frailty characteristics and clinical outcomes according to the new frailty-based outcome prediction model (Myeloma Risk Profile-MRP) in a UK real-world cohort of elderly newly diagnosed Myeloma patients

**DOI:** 10.1371/journal.pone.0262388

**Published:** 2022-01-11

**Authors:** Faouzi Djebbari, Alexandros Rampotas, Fotios Panitsas, Wen Yuen Lim, Charlotte Lees, Ismini Tsagkaraki, Ana Rita Gomes, Steve Prideaux, Lucia Chen, Catherine Prodger, Akhil Khera, Nicola Gray, Lauren Ellis, Gina Sangha, Toby A. Eyre, Sally Moore, Jaimal Kothari, Karthik Ramasamy

**Affiliations:** 1 Department of Clinical Haematology, Oxford University Hospitals NHS Foundation Trust, Oxford, United Kingdom; 2 Department of Haematology, Laiko General Hospital, Athens, Greece; 3 Royal Berkshire NHS Foundation Trust, Reading, United Kingdom; 4 Buckinghamshire Healthcare NHS Trust, Aylesbury, United Kingdom; 5 Great Western Hospitals NHS Foundation Trust, Swindon, United Kingdom; 6 Milton Keynes University Hospital NHS Foundation Trust, Milton Keynes, United Kingdom; 7 Wexham Park Hospital, Slough, United Kingdom; 8 Frimley Health NHS Foundation Trust, Frimley, United Kingdom; MD Anderson Center, UNITED STATES

## Abstract

The management of myeloma in the elderly is shifting its focus towards reducing the risk of under-treating fit patients and the risk of over-treating frail patients. Frailty assessment is required in this patient group in order to individualise treatment decisions. In addition to the proven prognostic values of the International Myeloma Working Group (IMWG) frailty score and the revised Myeloma Co-morbidity Index (R-MCI), a new easy-to-use frailty-based risk profile score (high-risk (i.e. frail), medium risk (i.e. intermediate-fitness) and low-risk (i.e. fit)) named Myeloma Risk Profile (MRP) was shown to be predictive of survival in the clinical trial setting. In this retrospective real-world study, we set out to evaluate the frailty characteristics and clinical outcomes according to the different MRP scoring algorithm categories (frail vs. intermediate vs fit), in a high risk cohort of elderly newly diagnosed myeloma patients treated with the fixed-duration triplet therapy VCD (bortezomib with cyclophosphamide and dexamethasone). Clinical outcomes included: reason for treatment discontinuation, overall response rate (ORR), overall survival (OS), progression-free survival (PFS), and adverse events (AEs). Out of 100 patients, 62 were frail, 27 were intermediate and 11 were fit, according to MRP scores. To enable meaningful comparisons between comparable numbers, subgroups analyses for ORR, OS, PFS, and AEs focused on frail (n = 62) versus intermediate or fit (n = 38) patients. The proportion of patients in each subgroup who were able to complete the planned course of treatment was (frail: 43.5% vs. intermediate or fit: 55.3%). A higher proportion in the frail subgroup discontinued therapy due to progressive disease (19.4% vs. 2.6%). Discontinuation due to toxicity was comparable across subgroups (14.5% vs. 15.8%), ORR in the total cohort was 75%, and this was comparable between subgroups (frail: 74.2% vs. intermediate or fit: 76.3%). There was a trend for a shorter median OS in the frail subgroup but without a statistical significance: (frail vs. intermediate or fit): (46 months vs. not reached, HR: 1.94, 95% CI 0.89–4.2, p = 0.094). There was no difference in median PFS between subgroups: (frail vs. intermediate or fit): (11.8 vs. 9.9 months, HR: 0.99, 95% CI: 0.61–1.61, P = 0.982). This cohort demonstrated a higher incidence rate of AEs in frail patients compared to those in the intermediate or fit group: patients with at least one any grade toxicity (85.5% vs. 71.1%), patients with at least one ≥G3 AE (37.1% vs. 21.1%). In conclusion, our study is to the first to evaluate clinical outcomes according to MRP in a high risk real-world cohort of patients treated exclusively with the proteasome inhibitor-based VCD therapy. Our study demonstrated a trend for worse OS in addition to worse AE outcomes in the frail group, but no difference in PFS with this fixed-duration therapy. MRP is an easy-to-use tool in clinical practice; its prognostic value was validated in the real-world in a large cohort of patients from the Danish Registry. Further evaluation of MRP in the real-world when continuous therapies are used, can further support the generalisability of its prognostic value in elderly myeloma patients.

## Introduction

Multiple myeloma (MM) is primarily a disease of the elderly with up to 44% of the UK’s newly diagnosed (NDMM) patients aged 75 and over [1]. The highest incidence rates in both males and females occur in those aged 85–89 years [1].

Achieving optimal myeloma outcomes in transplant-ineligible (TNE) NDMM is a challenge because patients can present with advanced age, organ dysfunction, co-morbidities, and frailty. Increased toxicity burden can drive dose reductions or early treatment discontinuation. In clinical practice, the focus is shifting towards reducing the risk of under-treating fit patients and the risk of over-treating frail patients [2]. Therefore, a structured frailty assessment is required in elderly myeloma patients, in order to devise individualised treatment plans [2].

The International Myeloma Working Group (IMWG) developed and validated a frailty score which is predictive of survival and toxicities [3, 4]. However, its routine application may be limited by time-constraints in a busy clinical setting. The revised Myeloma Co-morbidity Index (R-MCI) has also been shown to be a valid prognostic tool for overall survival (OS) in a large myeloma cohort [5]. The R-MCI is characterised by its accurate assessment of patients’ physical conditions and co-morbidities, the option to include disease cytogenetics, its value both in younger and older patients, in addition to its effective ability to identify at-risk patients [5].

More recently, The UK Myeloma Research Alliance (MRA) developed a frailty-based risk score named Myeloma Risk Profile (MRP), in TNE patients, using 3 risk profiles: high risk (frail), medium risk (intermediate-fitness), and low risk (fit) [6]. It utilises four widely used and readily available baseline patient and disease parameters: age, WHO performance status (PS), C-reactive protein (CRP), and myeloma International Staging system (ISS). The MRP model was shown to be predictive of OS [6]. The prognostic value of MRP was recently validated in a population-based study of 1377 TNE NDMM patients aged over 65 years, from the Danish National Myeloma Registry [7].

This retrospective study aims to use the MRP model to evaluate the frailty characteristics in a real-world cohort of TNE NDMM patients. This cohort included patients with advanced age, co-morbidities and high risk disease characteristics, who were consecutively treated with the proteasome inhibitor (PI)-based therapy VCD (bortezomib with cyclophosphamide and dexamethasone) within the UK’s Thames Valley Cancer Network (TVCN), outside of the clinical trial setting.

As an exploratory outcome, we also attempted to evaluate the prognostic value of MRP in this real-world cohort for survival and adverse events (AEs) according to the different MRP categories.

## Methods

### Study design and data collection

In this retrospective study, TNE NDMM patients are defined as those with a new diagnosis of symptomatic multiple myeloma which required initiation of systematic first line therapy, but who are not eligible for, and did not receive ASCT due to age and/or co-morbidities. All patients with TNE NDMM within the UK Thames Valley Cancer network treated with at least one cycle of VCD chemotherapy were eligible for inclusion. VCD is a 21 day regimen and is usually given for 6–8 cycles as follows: bortezomib 1.3mg/m^2^ subcutaneously (days 1,8,15), oral cyclophosphamide 500mg (days 1,8,15), and dexamethasone 20mg (days 1,2,8,9,15,16). All patients consented for retrospective review of their records at the point of treatment, and all patient records were anonymised at the point of analysis. Service evaluation approval was obtained from Oxford University Hospitals’ Haematology Clinical Governance Team, prior to starting the study. Data were censored on 01.01.2020. Data were retrospectively accessed by the myeloma research group between 01.01.2020 and 01.09.2020 to enable data collection.

Patients’ medical and chemotherapy records were used to collect baseline patient characteristics (age, sex, performance status (PS), co-morbidity Charlson Co-morbidity Index (CCI) score, anaemia, hypercalcaemia, renal impairment and baseline CRP). Baseline disease characteristics collected were: myeloma subtype, lactate dehydrogenase (LDH), myeloma International Staging System (ISS), and cytogenetics. High risk cytogenetics was defined as one or more of the following abnormalities in 20% of cells by fluorescence in situ hybridization (FISH): t(4;14), t (14;16), del(17p), t(14;20), 1q gain.

Treatment data collected were: number of cycles received, cumulative doses of bortezomib (mg/m^2^), of cyclophosphamide (in mg) and of dexamethasone (in mg), reasons for treatment discontinuation, and prophylactic anti-infective medication (antiviral, antifungal and PCP prophylaxis). VCD treatment plan is considered completed once optimal or best response agreed between patient and clinician is achieved, typically within 6–8 cycles on average.

### Calculation of the frailty MRP score and final inclusion of the cohort

The MRP (myeloma risk profile) score calculation and categorisation requires age, performance status, baseline CRP and ISS staging. Out of a total cohort of 158 patients treated with VCD, 58 patients were excluded: 24 had unknown CRP, 19 had unknown ISS, 14 had both ISS and CRP missing, and 1 patients had unknown PS and ISS. The final cohort, therefore, included 100 patients. In those patients who were eligible for inclusion in the final 100 cohort, VCD therapy was initiated between June 2012 and Dec 2018.

For each patient, the overall MRP score was calculated according to equation described in the original Lancet paper which first described this model, as per page 4 in the supplement document of the original paper [6]. Patients’ fitness was categorised according to the overall MRP score as follows: frail (score > - 0.0283), intermediate fitness (-0.256 ≤ score ≤ -0.0283), or fit (score < - 0.256) [6].

### Study endpoints

**Fig 1:**
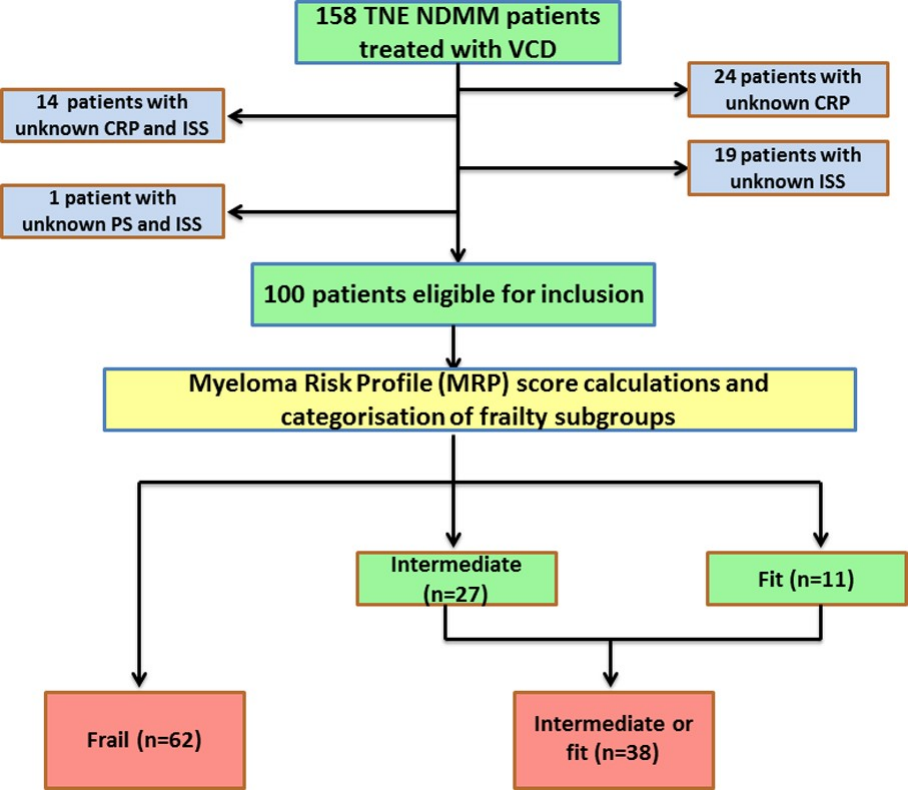
Inclusion/exclusion in the cohort and frailty score categories.

Out of 100 patients, 62 were frail, 27 were intermediate and 11 were fit. To enable meaningful comparisons in outcomes between comparable numbers, subgroups analyses focused on frail (n = 62) versus intermediate or fit (n = 38), Fig 1 (Inclusion/exclusion in the cohort and frailty score categories). Details on the number of treatment cycles and cumulative doses reported as follows: number of cycles (<6 vs. ≥6), cumulative bortezomib dose (≥26mg/m^2^ vs. <26mg/m^2^), cumulative cyclophosphamide dose (≥7000mg vs. <7000mg) and cumulative dexamethasone dose (>600mg vs. ≤600mg). These cut off values for cumulative doses of VCD components were specifically chosen because they represented the nearest dose to the median value in the total cohort, and they split the cohort equitably.

Response rates were evaluated in the total cohort and between frailty subgroups, according to the response assessment criteria of the International Myeloma Working Group (IMWG).

Progression-free survival (PFS) and overall survival (OS) were also evaluated between frailty subgroups (frail vs. intermediate or fit) as an exploratory outcome of the role of the MRP model in predicting outcomes in this real-world cohort.

PFS was evaluated as the time in months between initiation of VCD treatment and progressive disease (based on IMWG uniform response criteria) or start of second line therapy or death. OS was defined as time in months from initiation of VCD treatment to death from any cause. Common Terminology Criteria for Adverse Events (CTCAE) version 4.03 [8] was used to collect data on AEs attributed to VCD therapy.

### Statistical analyses

Descriptive statistics for quantitative variables are presented as median (interquartile range [IQR]). Descriptive statistics for categorical variables are presented as number (%). Time to event outcomes were evaluated and presented using the Kaplan-Meier method and reported as median (IQR). Time-to-event outcomes were compared between the different subgroups using unstratified log-rank tests and Cox regression analyses, with proportionality of hazards evaluated by visual assessment of “log-log” plots, and hazard ratios (HR) presented with 95% CI.

### Univariate (UVA) and multivariate (MVA) analyses for PFS and OS

Covariates with prognostic significance (p≤0.1) in univariate testing were included in the initial multivariate models of the corresponding outcome. Final models were arrived at by backward selection. Variables were retained in the final model if significant at the 5% level. No adjustment has been made for multiple testing.

We consider all UVA/MVA analyses exploratory and descriptive of our dataset. Analysis was done with STATA version 11.2 (StataCorp. 2009. Stata Statistical Software: Release 11. College Station, TX: StataCorp LP) and EZR [9].

## Results

### Patient, disease and treatment characteristics

Median follow-up (FU) of the overall cohort (n = 100) was 33.9 months (IQR 21.9–43.6, range 12.5–82.8 months). In the frail group, median FU was 33.9 months (IQR 21.9–42.2 months, range 13.4–57.4 months). In the Intermediate-fit cohort, median FU was 32.4 months, IQR 23.7–48.4 months, range 12.5–82.8 months). Follow-up did not differ between the two groups (Wilcoxon rank-sum p = 0.56).

The baseline patient, disease and treatment characteristics of the total cohort (n = 100) and by MRP subgroups (frail vs. intermediate or fit) are presented in Table 1. The median age (IQR) in the total cohort was 76 years (73–79), 63% had ISS III staging and 57.4% had high risk cytogenetic features (from a total of 61 patients with known data). Median number of treatment cycles was 7 (IQR 5–9).

**Table 1:** Baseline patient, disease and treatment characteristics for the total cohort, and for the different MRP subgroups (frail vs. intermediate or fit).

Baseline characteristics			Total cohort	Frailty subgroups		
			n = 100 (100%)			
				Frail	Intermediate or fit	P value
				n = 62(100%)	n = 38 (100%)	
**Patient**	Age (years): median (IQR)		76 (73–79)	77 (74–81)	74 (72–78)	0.0011^W^
	Sex	Male	53 (53%)	32 (51.6%)	21 (55.3%)	0.723^P^
		Female	47 (47%)	30 (48.4%)	17 (44.7%)	
	Performance Status	0	19 (19.2%)	0 (0%)	19 (50%)	<10^−3^ ^F^
		1	37 (37.4%)	22 (35.5%)	15 (39.5%)	
		2–3	44 (43.4%)	40 (64.5%)	4 (10.5%)	
	Co-morbidities (CCI score)*	Median (IQR)	4 (3–5)	4 (3–5)	3 (3–5)	
		CCI <5	64 (64.7%)	36 (59%)	28 (73.7%)	0.138^P^
		CCI ≥5	35 (35.4%)	25 (41%)	10 (26.3%)	
	Anaemia*	Yes	92 (92.9%)	58 (95.1%)	34 (89.5%)	0.423^F^
	Hypercalcaemia*	Yes	21 (21.2%)	17 (27.9%)	4 (10.5%)	0.046^F^
	Renal impairment	Yes	21 (21%)	14 (22.6%)	7 (18.4%)	0.62^P^
	CRP	Median (IQR)	6.3 (2–15.5)	6.8 (2.4–23.8)	4.8 (1–12.6)	0.1788^W^
**Disease**	MM subtype	Ig (G/A/M/D)	71 (71%)	42 (67.7%)	29 (76.3%)	0.075^F^
		Light chain	27 (27%)	20 (32.3%)	7 (18.4%)	
		Non-secretory	2 (2%)	0 (0%)	2 (5.3%)	
	Elevated LDH*	Yes	42 (58.3%)	29 (65.9%)	13 (46.4%)	0.102^P^
	ISS staging	1	6 (6%)	0 (0%)	6 (17%)	<10^−3^ ^F^
		2	31 (31%)	12 (21%)	16 (46%)	
		3	63 (63%)	44 (79%)	13 (37%)	
	Cytogenetics*	High risk (HR)	35 (57.4%)	20 (57.1%)	15 (57.7%)	0.966^P^
		Non-HR	26 (42.6%)	15 (42.9%)	11 (42.3%)	
**Treatment**	Number of VCD cycles	Median (IQR)	7 (5–9)	8 (5–9)	6 (5–9)	0.6682^W^
		<6	34 (34%)	21 (33.9%)	13 (34.2%)	0.972^P^
		≥6	66 (66%)	41 (66.1%)	25 (65.8%)	
	Cumulative bortezomib dose (mg/m^2^)	Median (IQR)	26 (19.5–34)	27.65 (18.5–35.1)	23.4 (19.5–32.3)	0.7594^W^
		<26mg/m^2^	49 (49%)	27 (43.6%)	22 (57.9%)	0.164^P^
		≥ 26mg/m^2^	51 (51%)	35 (56.5%)	16 (42.1%)	
	Cumulative cyclophosphamide dose (mg)	Median (IQR)	6850 (4300–9000)	6300 (3600–10500)	7500 (5250–9000)	0.4212^W^
		<7000mg	51 (51%)	35 (56.5%)	16 (42.1%)	0.164^P^
		≥7000 mg	49 (49%)	27 (43.6%)	22 (57.9%)	
	Cumulative dexamethasone dose (mg)	Median (IQR)	670 (440–960)	640 (380–960)	720 (380–960)	0.3602^W^
		<600mg	37 (37%)	26 (41.9%)	11 (29%)	0.192^P^
		≥ 600mg	63 (63%)	36 (58.1%)	27 (71.1%)	
	Anti-viral prophylaxis	Yes	99 (99%)	61 (98.4%)	38 (100%)	1 ^F^
	Anti-fungal prophylaxis	Yes	7 (7%)	6 (9.7%)	1 (2.6%)	0.247^F^
	PCP prophylaxis	Yes	57 (57%)	35 (56.5%)	22 (57.9%)	0.887^P^

Abbreviations: PS (performance status), CCI (Charlson co-morbidity index), MM (multiple myeloma), CRP (C-reactive protein), ISS (international staging system for MM), VCD (bortezomib with cyclophosphamide and dexamethasone), PCP (pneumocystis pneumonia).

*Number of patients with unknown data in the total cohort of 100 patients: 1 for CCI, 1 for anaemia, 1 for hypercalcaemia, 28 for LDH, 39 for cytogenetics. High risk cytogenetics is defined as one or more of the following features: t(4;14), t (14;16), del(17p), t(14;20), 1q gain. For P values,

^w^ superscript refers to Wilcoxon rank-sum p value,

^P^ superscript refers to Pearson chi square P value, and,

^F^ superscript refers to Fisher’s exact P value.

Patients in the frail subgroup were older (median age 77 vs. 74 years), had worse PS (≥2: 64.5% vs. 10.5%), a higher median CRP value (6.8 vs. 4.8), more patients with ISS III staging (79% vs. 37%), and a higher proportion of severely co-morbid patients according to Charlson Co-morbidity Index (CCI): (≥5: 41% vs. 26.3%). Median numbers of chemotherapy cycles as well as the cumulative doses of bortezomib, cyclophosphamide and dexamethasone were comparable between subgroups (Wilcoxon rank-sum).

### Treatment discontinuations

The proportion of patients in each subgroup who were able to complete the planned course of treatment was (frail: 43.5% vs. intermediate or fit: 55.3%). A higher proportion in the frail subgroup discontinued therapy due to progressive disease (19.4% vs. 2.6%). Discontinuation due to toxicity was comparable across subgroups (14.5% vs. 15.8%), Table 2.

**Table 2:** Reasons for discontinuation of first line VCD myeloma therapy in the total cohort, in MRP subgroups (frail vs. intermediate or fit) and in co-morbidity subgroups.

Reason for frontline VCD discontinuation	Total cohort (*n* = 100)	Frailty subgroups (total n = 100, 100%)			Co-morbidity subgroups*		
		Frail	Intermediate or fit	Fisher’s exact	CCI <5	CCI ≥ 5	Fisher’s exact
		n = 62 (100%)	n = 38 (100%)	P value	(n = 64) (100%)	(n = 35) (100%)	P value
				0.064			0.134
Course completion	48 (48%)	27 (43.5%)	21 (55.3%)		34 (53.1%)	14 (40.0%)	
Toxicities	15 (15%)	9 (14.5%)	6 (15.8%)		10 (15.6%)	5 (14.3%)	
Progressive disease	13 (13%)	12 (19.4%)	1 (2.6%)		8 (12.5%)	5 (14.3%)	
Death	7 (7%)	5 (8.1%)	2 (5.2%)		1 (1.6%)	5 (14.3%)	
Other**	14 (14%)	6 (9.7%)	8 (21%)		10 (15.6%)	4 (11.4%)	
NK	3 (3%)	3 (4.8%)	0 (0%)		1 (1.6%)	2 (5.7%)	

Data presented as % or n (%). Abbreviations: VCD (bortezomib with cyclophosphamide and dexamethasone), CCI (Charlson Co-morbidity Index). *The number of patients with unknown co-morbidity data in the total cohort of 100 patients is 1.

**Other reasons for VCD discontinuation include escalation to VTD due to high cytogenetic risk (in 2 patients), escalation to VTD due to poor response (1 patient), stable disease–switched treatment (in 3 patients), stable disease/plateau of response/inadequate response (in 3 patients), toxicity/stable disease (in 2 patients), patient’s decision to stop treatment (in 1 patient), stroke/best interest decision to stop treatment (in 1 patient), moved to another trust (1 patient).

### Response rates and survival outcomes

Overall response rate (ORR) in the total cohort was 75%, and this was comparable across frailty subgroups (frail: 74.2% vs. intermediate or fit: 76.3%), Table 3.

**Table 3:** Response rates to first line VCD myeloma therapy in the total cohort, in MRP subgroups (frail vs. intermediate or fit) and in co-morbidity subgroups.

Response to frontline VCD	Total cohort (*n* = 100)	Frailty subgroups (total n = 100, 100%)			Co-morbidity subgroups*		
		Frail	Intermediate or Fit	P value	CCI <5	CCI ≥ 5	P value
		n = 62 (100%)	n = 38 (100%)		(n = 64) (100%)	(n = 35) (100%)	
**ORR**	75 (75%)	46 (74.2%)	29 (76.3%)	Pearson chi square p = 0.812	52 (81.3%)	23 (65.7%)	Pearson chi square p = 0.085
**Best response**				Fisher’s exact p = 0.239			Fisher’s exact p = 0.061
≥ VGPR	49 (49%)	32 (51.6%)	17 (44.7%)		34 (53.1%)	15 (42.8%)	
PR	26 (26%)	14 (22.6%)	12 (31.6%)		18 (28.1%)	8 (22.9%)	
SD	15 (15%)	7 (11.3%)	8 (21%)		10 (15.6%)	5 (14.3%)	
PD	8 (8%)	7 (11.3%)	1 (2.6%)		1 (1.6%)	6 (17.1%)	
NK	2 (2%)	2 (3.2%)	0 (0%)		1 (1.6%)	1 (2.9%)	

Data presented as % or n (%). Abbreviations: VCD (bortezomib with cyclophosphamide and dexamethasone), CCI (Charlson Co-morbidity Index), ORR (overall response rate), CR (complete Response), VGPR (very good partial response); PR (partial response), SD (stable disease), PD (progressive disease), NK (unknown). *The number of patients with unknown co-morbidity data in the total cohort of 100 patients is 1.

**Fig 2:**
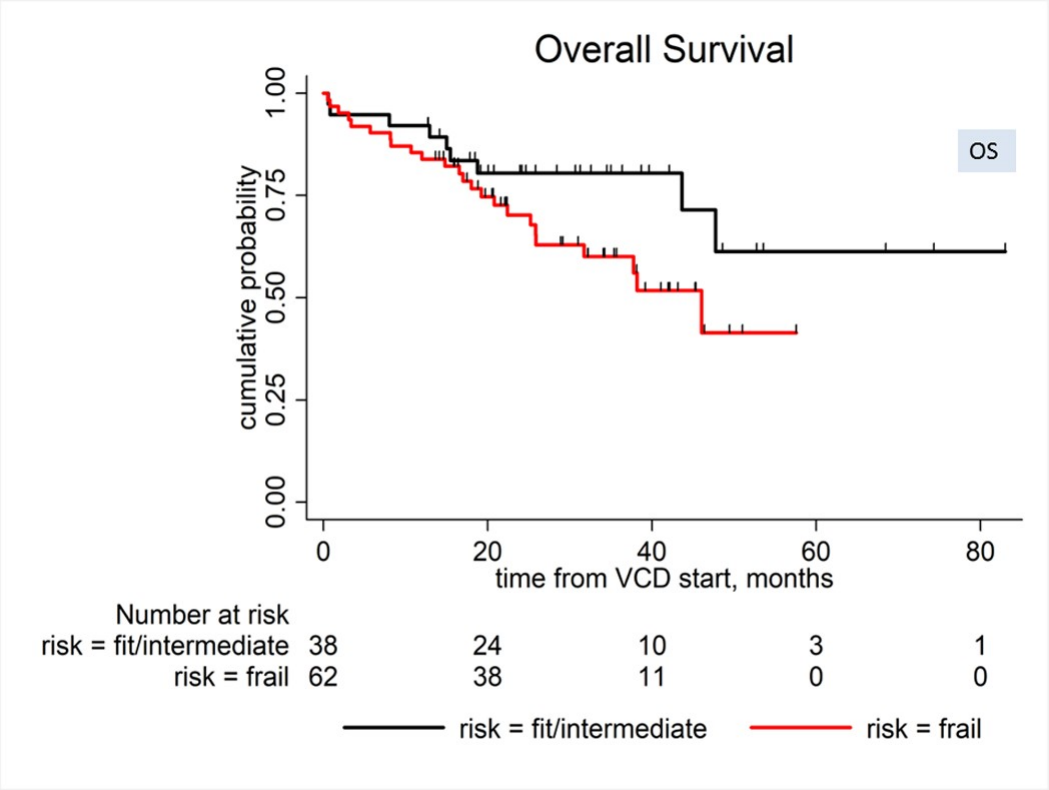
Overall survival (OS) by frailty (frail vs. intermediate or fit): (46 months vs. not reached, HR:1.94, 95% CI 0.89–4.2, p = 0.094).

**Fig 3:**
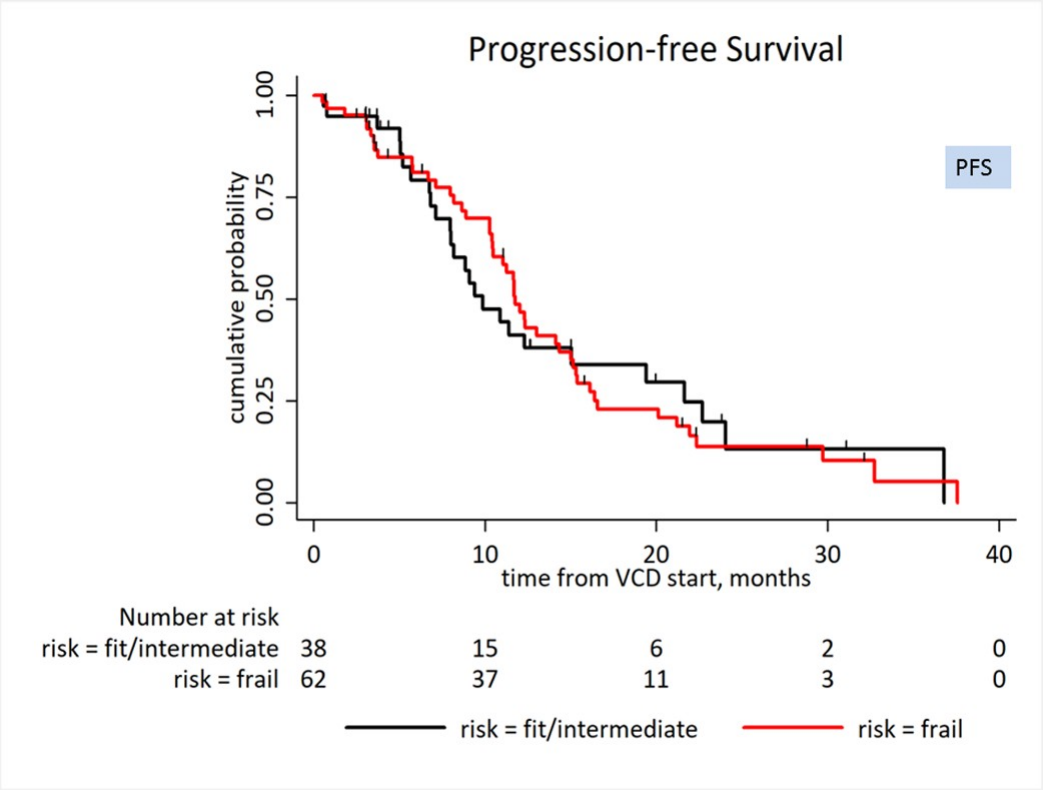
Progression-free survival (PFS) by frailty (frail vs. intermediate or fit): (11.8 vs. 9.9 months, HR: 0.99, 95% CI: 0.61–1.61, P = 0.982).

There was a trend for a shorter median OS in the frail subgroup but without a statistical significance: (frail vs. intermediate or fit): (46 months vs. not reached, HR: 1.94, 95% CI 0.89–4.2, p = 0.094), Fig 2 (Overall survival (OS) by frailty (Frail vs. Intermediate or Fit)). The predictive ability of the MRP frailty scoring for OS was low in this cohort (Harrell’s C 0.562). There was no difference in median PFS between subgroups: (frail vs. intermediate or fit): (11.8 vs. 9.9 months, HR: 0.99, 95% CI: 0.61–1.61, P = 0.982), Fig 3 (Progression-free survival (PFS) by frailty (Frail vs. Intermediate or Fit)).

To further explore factors associated with PFS and OS outcomes besides MRP frailty, we conducted univariate (UVA) and multivariate (MVA) analyses. MVA model only included covariates with a prognostic value p≤0.1 in UVA. Results are presented in Table 4. By UVA, the cumulative cyclophosphamide dose (≥7000mg) was the only factor to have a statistically significant association with PFS (p = 0.008). In the absence of other variables of prognostic value, MVA PFS analysis was not performed.

**Table 4:** Univariate (UVA) and multivariate (MVA) analyses for PFS and OS.

Outcome	Characteristic	Univariate analysis			Multivariate analysis		
		Hazard ratio	95% CI	P-value	Hazard ratio	(95% CI)	P-value
**PFS** ^**+**^	Age (years): (<75 vs. ≥75)	1.19	0.74–1.93	0.47	-	-	-
	Performance status (2–3 vs. 0–1)	0.88	0.55–1.41	0.59	-	-	-
	Co-morbidity CCI (≥5 vs. <5)	1.2	0.74–1.95	0.47	-	-	-
	LDH (normal vs. elevated)	1.11	0.64–1.93	0.71	-	-	-
	ISS staging (III vs. <III)	1.015	0.6–1.73	0.96	-	-	-
	MRP (frail vs. intermediate-fit)	0.99	0.61–1.61	0.98	-	-	-
	Cytogenetics (HR vs. non-HR)	0.94	0.51–1.71	0.83	-	-	-
	Number of VCD cycles (<6 vs. ≥6)*	0.94	0.42–2.11	0.88	-	-	-
	Cum bort dose (<26 vs. ≥ 26mg/m^2^)*	0.87	0.48–1.56	0.64	-	-	-
	Cum cyclo dose (<7 vs. ≥ 7g)*	2.08	1.21–3.57	0.008	-	-	-
	Cum dex dose (<600 vs. ≥ 600mg)*	0.93	0.52–1.67	0.82	-	-	-
**OS** ^**+**^	Age (years): (<75 vs. ≥75)	1.55	0.75–3.2	0.24	-	-	-
	Performance status (2–3 vs. 0–1)	1.37	0.68–2.75	0.38	-	-	-
	Co-morbidity CCI (≥5 vs. <5)	1.90	0.94–3.79	0.08	1.86	0.88–3.93	0.106
	LDH (normal vs. elevated)	1.04	0.43–2.52	0.93	-	-	-
	ISS staging (III vs. <III)	2.92	1.18–7.24	0.012	2.32	0.88–6.15	0.09
	MRP (frail vs. intermediate-fit)	1.94	0.89–4.2	0.094	1.66	0.66–4.19	0.28
	Cytogenetics (HR vs. non-HR)	0.9	0.35–2.28	0.82	-	-	-
	Number of VCD cycles (<6 vs. ≥6)*	1.22	0.53–2.83	0.64	-	-	-
	Cum bort dose (<26 vs. ≥ 26mg/m^2^)*	0.92	0.41–2.1	0.85	-	-	-
	Cum cyclo dose (<7 vs. ≥ 7g)*	1.09	0.5–2.4	0.82	-	-	-
	Cum dex dose (<600 vs. ≥ 600mg)*	0.66	0.26–1.65	0.37	-	-	-

*Parameters of treatment cycles and cumulative doses were included in this analysis using a 6-month landmark analysis in order to reduce the risk of survivorship bias. ^+^The MVA model only included covariates with a prognostic value (p≤0.1 in UVA).

ISS staging was the only factor by UVA to have a statistically significant association with OS (p = 0.012). Severely co-morbid patients (CCI≥5) and those in the frail MRP subgroup, showed a trend for a worse OS but without statistical significance (p = 0.08 and p = 0.094, respectively). The MVA OS analysis included ISS staging, co-morbidities and frailty, none of which was shown to be independently associated with OS, although there was a trend for better OS for patients with ISS <III, albeit without a statistical significance (p = 0.09).

### Adverse events (AEs)

Data on AEs is fully presented in Table 5. The total number of any grade AEs in the total cohort was 222, experienced by a total of 80 patients. The median (range) of any grade AEs per patient in the total cohort was 2 (1–7). The total number of all ≥G3 AEs in the total cohort was 43, experienced by a total of 31 patients. This study demonstrated a higher incidence of AEs in frail patients compared to those in the intermediate or fit group.

**Table 5:** Toxicities associated with VCD therapy in the total cohort.

Body System	AE name	Total cohort evaluable for AEs, n = 100 (100%)			
		Incidence (number of events)		% of patients	
		Any grade	≥G3	Any grade	≥G3 =
Blood and lymphatic system disorders	Neutropenia	15	4	15%	4%
	Lymphopenia	1	0	1%	0%
	Thrombocytopenia	20	2	20%	2%
	Anaemia	17	9	17%	9%
Infections	Infections	48	17	36%	14%
CNS disorders	Peripheral neuropathy	38	4	38%	4%
Gastrointestinal disorders	Constipation	12	0	12%	0%
	Diarrhoea	9	2	9%	2%
	Nausea	9	0	9%	0%
	Abdominal pain	1	0	1%	0%
	Gastritis	2	0	2%	0%
	Mucositis	1	0	1%	0%
Psychiatric AEs	Agitation	2	0	2%	0%
	Confusion	2	0	2%	0%
	Insomnia	1	0	1%	0%
	Psychosis	1	1	1%	1%
	Depression	2	0	2%	0%
Cardiac AEs	Hypotension	2	0	2%	0%
	Syncope	2	2	2%	2%
	Chest pain	1	0	1%	0%
	Atrial fibrillation	1	0	1%	0%
Metabolism and nutrition AEs	Hyperglycaemia	6	0	6%	0%
	Cushingoid syndrome	1	0	1%	0%
	SIADH	1	1	1%	1%
	Osteoporosis	1	0	1%	0%
	Anorexia	1	1	1%	1%
Musculo-skeletal AEs	Synovitis/arthopathy	1	0	1%	0%
	Peripheral oedema	5	0	5%	0%
Immune reactions	Allergic reaction	1	0	1%	0%
	Rash	2	0	2%	0%
Renal AEs	Acute kidney injury	1	1	1%	1%
Respiratory AEs	Pneumonitis	1	1	1%	1%
Vascular AEs	Haematoma	1	0	1%	0%
Eye AEs	Blurred vision	1	0	1%	0%
Ear AEs	Hearing impairment	3	0	3%	0%
General AEs	Fatigue	9	1	9%	1%

In the frail group, 53/62 (85.5%) and 23/62 (37.1%) experienced at least one any grade, and at least one ≥G3 AE respectively, with median number of episodes per patient (ranges) of 2(1–7) and 1 (1–4). In the same group, 8/62 (12.9%) experienced at least one ≥G3 haematological toxicity with a median number of episodes (range) of 1 (1–3).

In the intermediate or fit group, 27/38 (71.1%) and 8/38 (21.1%) experienced at least one any grade, and at least one ≥G3 AE respectively, with medians (ranges) of 3 (1–6) and 2 (1–3). In the same group, 3/38 (7.9%) experienced at least one ≥G3 haematological toxicity with a median number of episodes (range) of 1 (1–2).

## Discussion

The choice of VCD therapy made by clinicians to treat this elderly newly diagnosed myeloma patient group in this UK cancer region is often driven by high risk/aggressive disease and renal presentation, instead of other available therapeutic alternatives such as attenuated cyclophosphamide with thalidomide and dexamethasone (CTDa). Access to lenalidomide with dexamethasone in this patient group (as per FIRST trial) was not available in UK routine practice until 2019.

The MPR risk scores are easy to calculate and can be readily implemented in clinical practice. The prognostic value of MRP was validated in a population-based study of 1377 TNE NDMM patients from the Danish National Myeloma Registry, which confirmed that the MRP is a robust and valuable risk assessment tool for patients aged over 65 years [7]. Median OS was (low risk: 55 vs. medium risk: 35.9 vs. high risk: 13.9 months, P<0.0001), and median PFS was (low risk: 21.7 vs. medium risk: 16.6 vs. high risk: 10.3 months, P<0.0001) [7].

However, there remains an ongoing debate on whether the current frailty assessments, such as MRP, are in need of further harmonisation prior to implementation into myeloma treatment decision-making in routine care, such as the inclusion of biomarkers for ageing [10], and other parameters reflective of baseline patient-related characteristics (e.g. co-morbidities, functional impairment).

In this study, we have shown a trend for worse OS in frail patients, which is consistent with MRP study, but without statistical significance. Our ability to assess the prognostic value of the MRP for survival was limited by the smaller patient numbers in the different MRP categories. This is to say that data fragmentation may have led to insufficient power to assess the prognostic value.

In addition, we focused mostly on describing frailty characteristics and outcomes in this real-world according MRP, and therefore it was not our primary aim to assess the prognostic value of MRP for survival, which was previously validated by the Danish Registry, and which we considered in this study as an exploratory outcome.

Our study is the first to evaluate frailty outcomes using MRP in the real-world in elderly newly diagnosed myeloma patients treated exclusively with a proteasome-inhibitor-based therapy. In addition, this study adds to the literature on MPR by evaluating its prognostic value for AEs through our comprehensive AE analysis in this high risk myeloma cohort.

Our study is limited by its relatively small sample size, its retrospective, non-randomised nature with the inherent possibility of unmeasured confounding factors, patient selection bias, the potential for medical chart misinterpretation and underreporting of toxicities.

Despite these limitations, this study has clearly demonstrated the high prevalence of frailty in the real-world (62%), according to the MRP model. This is significantly more than 46.4% high risk patients reported in the Danish Registry study [7], although this comparison is limited by the small sample size of our cohort. We also demonstrated a higher treatment toxicity burden in the frail MRP subgroup.

Our PFS results did not correlate with the UK MRA data which demonstrated that MRP is also predictive of PFS (high risk patients achieving a statistically inferior PFS) [6]. However, the lack of PFS difference may be explained by the presence of confounders, the high risk myeloma characteristics of this total cohort (ISS III in 63%, high risk cytogenetics in 57.4% of patients), in addition to the significant co-morbidity burden (median CCI: 4, CCI ≥5: 35.4%), and the low number of treatment cycles used with fixed duration therapy (FDT) strategy.

The myeloma treatment landscape for TNE NDMM patients is shifting towards the continuous therapy (CT) strategy, such as continuous lenalidomide and dexamethasone as per FIRST trial [11], continuous daratumumab with lenalidomide and dexamethasone as per MAIA trial [12], and continuous daratumumab with bortezomib, melphalan and prednisolone as per ALYCONE trial [13]. We envisage that CT will provide survival benefit to this patient group and will lead to an extended PFS. Further evaluation of MRP when these CT regimen are used, can further support the generalisability of its prognostic value in elderly myeloma patients.

## Conclusion

Our study is the first real-world study to evaluate clinical outcomes according to MRP in a real-world cohort of elderly newly diagnosed myeloma patients treated exclusively with a proteasome inhibitor-based therapy VCD. Our study demonstrated a trend for worse OS in addition to worse AE outcomes in the frail group compared to less frail patients, but no difference in PFS when fixed-duration VCD was used. MRP is an easy-to-use tool in clinical practice; its prognostic value was validated in the real-world in a large cohort of patients from the Danish Registry. Further evaluation of MRP in the real-world when continuous therapies are used, can further support its prognostic role in elderly myeloma patients.
